# Practical Strategies for Stable Operation of HFF-QCM in Continuous Air Flow

**DOI:** 10.3390/s130912012

**Published:** 2013-09-09

**Authors:** Alexander Wessels, Bernhard Klöckner, Carsten Siering, Siegfried R. Waldvogel

**Affiliations:** 1 Chemische Institute, Abteilung Elektronik, Rheinische Friedrich-Wilhelm-Universität Bonn, Gerhard-Domagk-Str. 1, Bonn D-55121, Germany; E-Mails: awessels@uni-bonn.de (A.W.); b.kloeckner@uni-bonn.de (B.K.); 2 Institut für Organische Chemie, Johannes Gutenberg-Universität Mainz, Duesbergweg 10-14, Mainz D-55128, Germany; E-Mail: siering@uni-mainz.de

**Keywords:** quartz crystal microbalance, high fundamental frequency, allan deviation, turbulences, laminar flow element, acceleration sensitivity, temperature gradient

## Abstract

Currently there are a few fields of application using quartz crystal microbalances (QCM). Because of environmental conditions and insufficient resolution of the microbalance, chemical sensing of volatile organic compounds in an open system was as yet not possible. In this study we present strategies on how to use 195 MHz fundamental quartz resonators for a mobile sensor platform to detect airborne analytes. Commonly the use of devices with a resonant frequency of about 10 MHz is standard. By increasing the frequency to 195 MHz the frequency shift increases by a factor of almost 400. Unfortunately, such kinds of quartz crystals tend to exhibit some challenges to obtain a reasonable signal-to-noise ratio. It was possible to reduce the noise in frequency in a continuous air flow of 7.5 m/s to 0.4 Hz [*i.e.*, σ(τ) = 2 × 10^−9^] by elucidating the major source of noise. The air flow in the vicinity of the quartz was analyzed to reduce turbulences. Furthermore, we found a dependency between the acceleration sensitivity and mechanical stress induced by an internal thermal gradient. By reducing this gradient, we achieved reduction of the sensitivity to acceleration by more than one decade. Hence, the resulting sensor is more robust to environmental conditions such as temperature, acceleration and air flow.

## 1. Introduction

When G. Sauerbrey developed the “Sauerbrey equation” [Equation (1)] in 1959, he was far away from developing sensitive mobile sensors to detect airborne analytes [1]:

(1) $$\Delta f=\frac{2\cdot {f_{0}}^{2}}{\text{A}\sqrt{\rho_{q}\mu_{q}}}\Delta m$$

This relation between the variation of the oscillating mass (*Δm*) and the shift of resonance frequency (*Δf*) of fundamental quartz crystal oscillators is also valid for thin films with a uniform mass distribution [2,3]. It depends on the active quartz crystal area *A*, the density ρ_q_ and the shear modulus μ_q_ of the quartz. Currently, there are a few fields of application using quartz crystal microbalances (QCM) [4], as well as highly dynamic research [5]. The typical setup of such QCM sensors follows the design principle introduced by King [6], using a chemical sensing layer on top of a QCM. By various kinds of chemical/physical interaction, the target analytes from the surrounding are bound to the sensitive layer. The resulting mass change causes a variation of the oscillation frequency.

**Figure 1.**
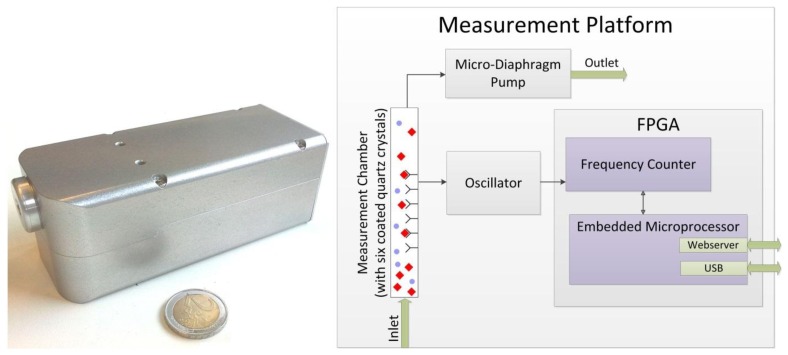
Prototype of the handheld measurement platform and schematic description of the measurement set-up.

Different from other approaches (e.g., [7–9]) we focus on the online detection of analytes in an airstream without using a preconcentrator. As real-time tracing of very dilute specimens is very desirable, a high sensitivity (in terms of high readout per unit analyte) of the whole sensor is pivotal. This can be achieved by tuning both the chemical and the engineering parts. Our development and selection of appropriate affinity materials for the chemical interaction has been described elsewhere [10,11]. The general setup is depicted in Figure 1.

**Figure 2.**
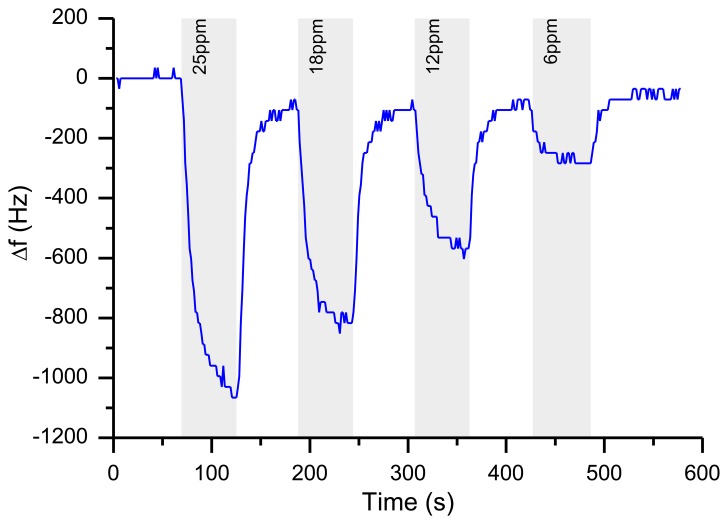
Typical curve for the detection of various TATP concentrations by a polyphenylene dendrimer; gray shaded areas represent time slots during which the analyte was added.

Six differently coated QCMs and an atmosphere containing the analyte are brought into contact in a housing (typically made of aluminum). For continuous measurements, this atmosphere is delivered either by slight pressure to the chamber (outlet is open to atmosphere, for calibration of quartzes, “closed setup”) or by suction using a pump at the outlet (for use as a mobile analyzer, “open system”). A typical frequency plot for an airborne analyte shows concentration dependent frequency shifts as depicted in Figure 2.

In this paper, we report on the various engineering strategies that were followed to improve the sensor resolution including signal processing and stream optimization.

### 1.1. Increasing the Sensitivity

There are discussions about the correct definition of “sensitivity” when coming to quartz oscillators.

The most simple is the absolute frequency shift in Hz/ppm analyte, assuming an almost linear relationship for sufficiently small concentration ranges. For comparison of QCM systems operating at different frequencies, the relative frequency shift (Δf/f_0_) may be more reasonable. However, as these devices exhibit a significant noise, the signal-to-noise ratio should be regarded as well.

Following Equation (1), a most practical way to increase the absolute frequency shift of a QCM device is the employment of a quartz crystal oscillator with higher operational frequencies. This can be achieved by higher fundamental frequencies, overtones of common quartz crystals or using completely differently acoustic sensor techniques (*i.e.*, SAW, FBAR). As discussed by Vig [12] several factors (aging, Q-factor, accuracy) will influence more strongly high-frequency quartzes and diminish the better theoretical absolute frequency shift. Yet, there are today even 8 GHz thin film oscillators [13] and several other reported systems in the range >100 MHz.

In this report, we will describe our approaches to build a QCM sensor based on commercially available high frequency quartz oscillators. As shown in Figure 2, the high frequency of the quartz crystal yields a high sensitivity (in terms of an absolute frequency shift). The specific frequency shift derived from this is approximately 35 Hz/ppm TATP. Special attention was given to meet challenges from environmental influences caused by the use of the system in a mobile fashion.

## 2. Measurement Setup

### 2.1. High Frequency Fundamental Quartz

**Figure 3.**
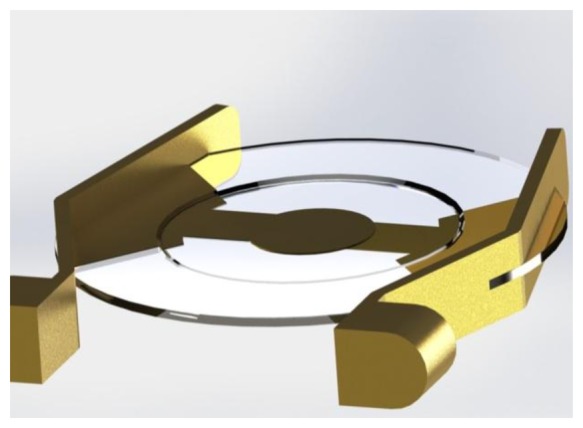
Schematic view of a high fundamental frequency quartz oszillator (diameter of the quartz disk approx. 5 mm).

The oscillators for this study (195 MHz fundamental frequency) were acquired from KVG Quartz Crystal Technology GmbH, Neckarbischofsheim, Germany. Due to their “inverted mesa” geometry, these devices exhibit a reasonable mechanical stability (Figure 3).

The quality-factor (Q-factor) is a measure for the quality of the quartz oscillator and is defined as the ratio of stored energy to dissipated energy. It is inversely proportional to the random fractional frequency fluctuations or short-term instabilities of a quartz oscillator. Therefore the Q-factor is an important factor for the signal-to-noise ratio (SNR) of a measurement. According to Warner [14] the theoretically maximum attainable Q-factor for AT-cut crystals should be in the range of 1.6 × 10^6^ for a 10 MHz quartz oscillator, whereas the theoretical maximum Q-factor for a 195 MHz quartz oscillator is limited to 8.2 × 10^4^. Due to additional losses this theoretical value is not reached in practice, especially when the quartz is not hermetically sealed like in a QCM application. Commonly the Q-factor varies from 10^2^ to 4 × 10^5^ in air [14,15]. The 195 MHz HFF-Quartz crystals are specified with a Q-factor of 6 × 10^3^ to 2.5 × 10^4^ (*cf.* Figure 3). Under atmospheric conditions we measured a Q-factor in the range of 1 × 10^4^ to 2 × 10^4^.

**Figure 4.**
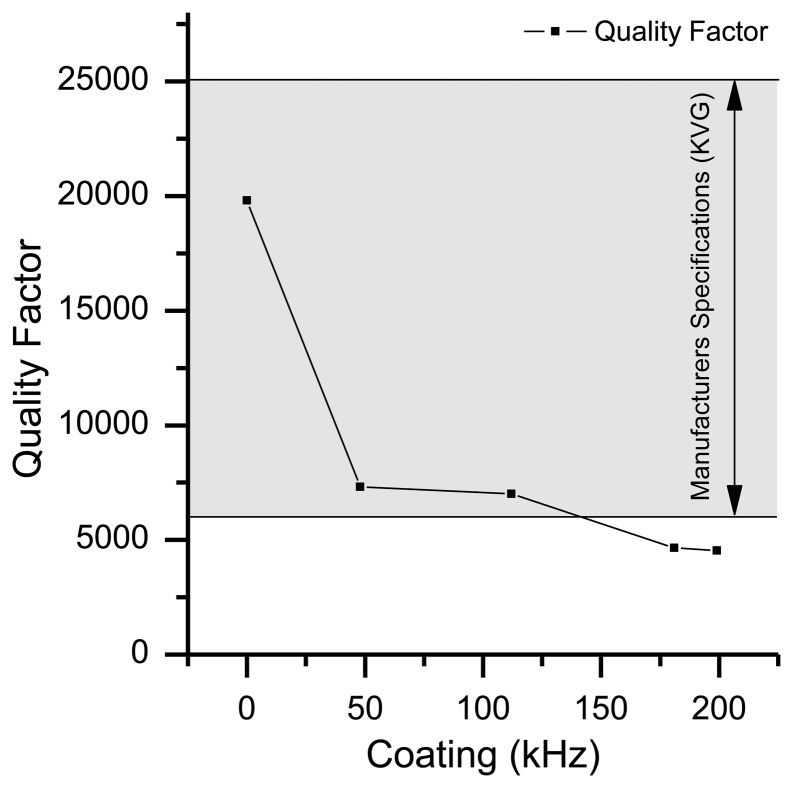
Decay of the Q-factor of a resonator with increasing coating thickness.

In order to evaluate the effect of coating to the Q-factor of quartz, the film thickness of a series of quartzes was increased iteratively. Figure 4 depicts the effect of coating on the Q-factor of one quartz crystal [assuming a homogeneous distribution of the affinity material on the oscillator surface, the film thickness is proportional to the mass difference Δ*m* upon coating, which again is proportional to the frequency shift by Equation (1), therefore, film thickness is given in kHz as in this report]. As anticipated, coating of the crystal reduces the Q-factor with the film thickness. In this work we used a coating of 50 kHz (corresponding to a deposited mass of 10 ng) which leads to a Q-factor of approximately 7∼8 × 10^3^. Compared to standard sealed quartz crystals with a Q-factor in the range of 10^3^ to 10^6^ this is still an acceptable value [15].

As recommended by the IEEE subcommittee of frequency we used the Allan deviation *σ*(*τ*) with a gate time of τ as SNR measure in the time domain [16]. According to Vig *et al.* [12] a Q-factor of 8 × 10^3^ would result in a minimum *σ*(*τ*) of 1.25 × 10^−10^. However, typical QCM systems have minimum Allan deviations in the range of 10^−6^ to 10^−8^ [15]. This is due to limitations of the electronic set-up and environmental conditions like temperature, acceleration of the handheld device, atmospheric pressure and air flow hard to realize. Therefore, the main goal of this work was to reduce the influence of environmental conditions to achieve a high signal to noise ratio.

### 2.2. Oscillator and Counter Description

Six oscillator circuits are realized as a Collpitts Oscillator by using the IC Max2620. This IC shows low phase noise and integrated buffers avert frequency pulling due to load-impedance changes.

Counting the frequency of these six oscillators can be done by using a simple “forward” counter. Due to the ±1 count error, the resulting relative frequency error is ±5 × 10^−9^ for a 195 MHz oscillator with a fixed gate time of 1 s. Another approach to count the frequency is a reciprocal counter. It enables real time control of the gate time and can increase the resolution significantly. If the frequency of the reference clock *f_ref_* is much higher than the signal *f_meas_* to be counted, the ±1 counting error for the implemented reciprocal counter can be calculated as:

(2) $$e(\tau )=\pm \frac{f_{\text{meas}}}{{f_{\text{ref}}}^{2}}\times \frac{1}{\tau }$$

To achieve *f_meas_*≪*f_ref_*, the frequency of the crystal oscillators has to be converted into a low frequency. This is often done by analog mixing (e.g., [17]). Unfortunately this needs high design efforts resulting in larger devices compared to forward counters with a fixed gate time.

Another approach is the use of digital mixing as it was done by Shankar *et al.* [18] or Bruckenstein *et al.* [19]. They used D-Flip-Flops as digital mixer by discrete circuit elements or by using a silicon-on-insulator process.

**Figure 5.**
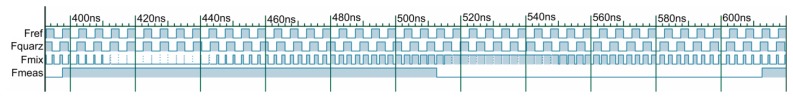
Schematic depiction of the digital mixing process.

We have implemented the digital mixer into an onboard FPGA. This enables a simpler implementation due to simulation and debugging of the programmable hardware. Digital ports of the FPGA are triggered by the rising edge of the sinusoidal analog oscillator signal. Therefore the signal is completely processed inside the FPGA. The mixer was formed by an XOR-Gate, which is the ideal digital representation of a gilbert cell in the analog domain. The frequency of the quartz oscillator *f_Quartz_* ≈ 195 MHz is mixed with a reference clock *f_Ref_* = 200 MHz [with *σ*(1 s) ≈ 9 × 10^−12^]. The resulting spectrum of the signal includes the frequencies *f_Ref_* − *f_Quartz_* and *f_Ref_* + *f_Quartz_*. To eliminate unwanted frequencies like *f_Ref_* + *f_Quarz_* the resulting signal is filtered by an FIR-Filter of 90th order. This is why the output of the filter has a delay of 90 clock cycles which is insignificantly small for a counter with a gate time in the range of ms (Figure 5).

With *f_Ref_* = 200 MHz and *f_meas_* = *f_Ref_* − *f_Quartz_* = 5 MHz the counting error (Equation (2)) can be calculated as:

(3) $$e(\tau )=\pm \frac{1.25\times 10^{-10}}{\tau }$$

Compared with the forward counter the counting error can be reduced from *e* = ±5 × 10^−9^ to *e*(1 s) = ±1.25 × 10^−10^ by using the digital mixer implemented into a FPGA.

## 3. Experimental Results

### 3.1. Signal-to-Noise Behavior without Airflow

Using the described measurement set-up a handheld measurement platform with a size of just 150 × 55 × 50 mm was realized (Figure 1). In order to evaluate the Allan deviation for this setup, the reciprocal counter was set in a way, that the given number of oscillations is complete after approx. 20 ms. By use of two parallel counters, it was ensured that each clock cycle was considered and the dead time was zero. Frequency values were recorded continuously over a time period of 2,500 s. For the Allan analysis it was assumed that every measurement represents 20 ms. The gate time as indicated here therefore represents multiples of 20 ms.

**Figure 6.**
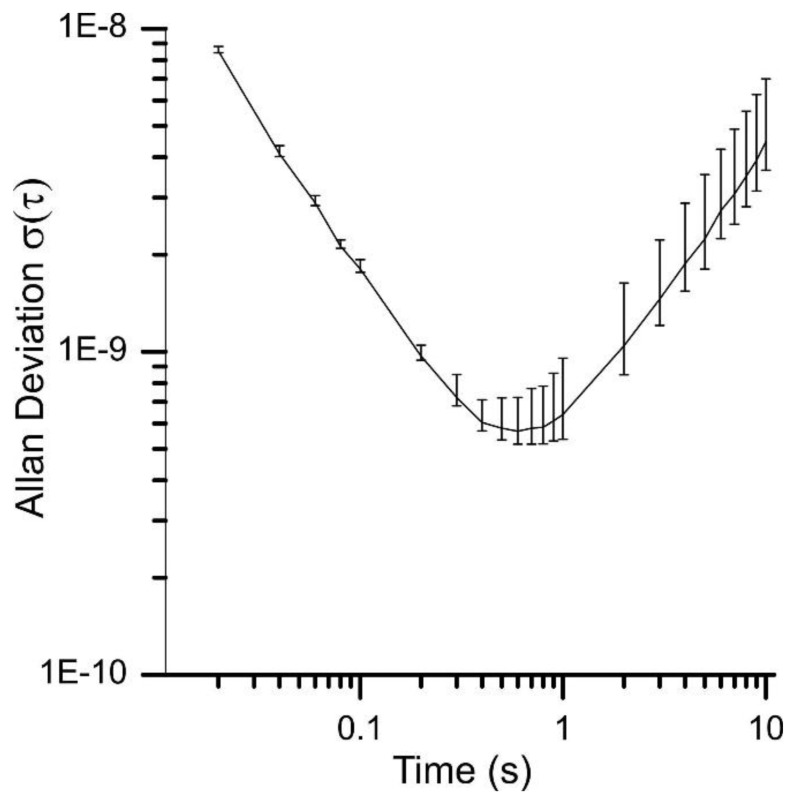
Allan deviation and errors of Quartz 1 just after the startup of the device (90% confidence interval).

Therefore the Allan deviation can be calculated for different gate times under the same environmental conditions. However, adding the measurements reduces the number of values for the calculation of the Allan deviation. This is why the error increases with the calculated gate time as it can be depicted from Figure 6. For simplification the error bars are not depicted in the following Allan plots. Nevertheless they show a similar error as shown in Figure 6.

The subsequent Allan plot show three quartz crystals: *Quartz 1* is an uncoated crystal, *Quartz 2* and *Quartz 3* are coated with two different compounds (50 kHz “film thickness” each). Among fabrication variation between individual quartzes, the difference between the shown results is attributed to the viscoelastic properties and the mechanical coupling of the different compounds to the quartz surface. As this report does not focus on the variation of quartz behavior upon different coatings, the three different quartzes were just investigated to expand the sample range and to show that the discussed effects are of more general nature.

**Figure 7.**
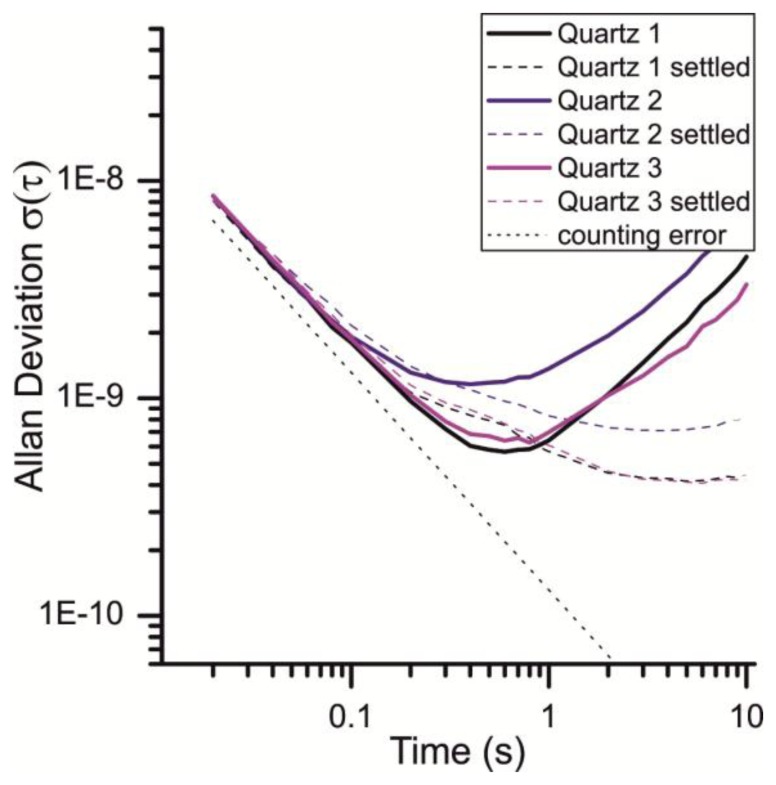
Allan deviation under atmospheric pressure at the beginning of a measurement and after 8 h operation time.

As expected the white noise, which is mainly caused by the ±1 counting error, increases for shorter gate times. With an increasing gate time the random walk, exceptionally caused by the warm up behavior, becomes more significant. With the runtime the device reaches its thermal equilibrium which is why the random walk is reduced. Figure 7 illustrates the difference of the Allan deviation measured just after the startup of the device and 8 h after. It shows that the Allan deviation of a quartz can vary greatly for different measurements and gate times greater than 0.8 s. To have a runtime independent signal-to-noise ratio, a gate time of 0.2 to 0.8 s seems to be the optimal choice for this device.

With these gate times, an Allan deviation of 6 × 10^−10^ to 2 × 10^−9^ is achieved in this system (Figure 7). This is remarkably close to the theoretical limit of 1.25 × 10^−10^ (vide supra). It should be noted that the oscillators are not in a sealed housing and still yield this excellent SNR. This can be measured without airflow, but under atmospheric pressure. Therefore the measurement platform is a good compromise of small signal to noise ratio, sensitivity (in terms of a frequency shift in Hz/ppm) and size. However, further design efforts can be made to reduce the counting error in order to reduce the white noise of the measurement.

### 3.2. Influence of Pressure and Turbulences

With these results in hands, the system was pushed further to the “real world”, *i.e.*, into an air stream. The influence of the atmospheric pressure on QCM frequency was well analyzed in different reports by Kokobun *et al.* [20]. Atmospheric pressure can produce a deformation of the crystal which can change the capacity and cause mechanical stress.

**Figure 8.**
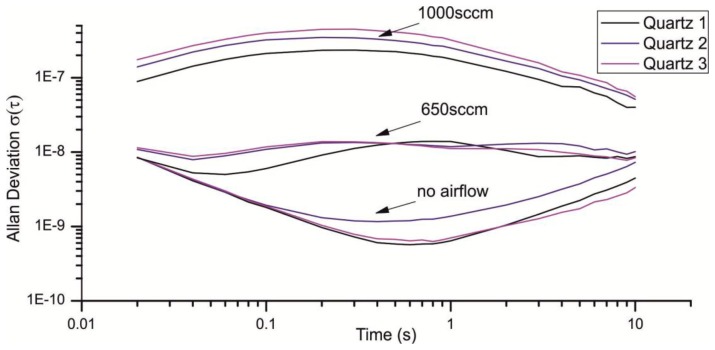
Allan deviation for different flow rates. The Allan deviation of 1,000 sccm and 650 sccm was measured at least 2 h after the device startup, whereas the Allan deviation without airflow was taken just after the startup.

However, in an online detector with a stream of air passing the quartzes, short-term pressure fluctuations might have the most relevant influence on signal stability. In our sensor system, the double-headed micro diaphragm pump NMP015 manufactured by KNF Neuenberger (Freiburg, Germany) with a maximal air flow of 2,200 sccm (standard cubic centimeters per minute) was used. The array of quartz crystals is located in a measurement chamber with 13 mm diameter. Figure 8 shows the resulting Allan deviation at different flow rates. For a flow rate of 0.08 m/s (650 sccm) the Allan deviation increases by more than one decade, for a flow rate of 0.13 m/s (1,000 sccm) to more than 10^−7^.

Turbulences in the airflow might be a source of noise in frequency. To get an idea about the influence of these turbulences the system was modeled and analyzed with SolidWorks^®^ [21]. An accurate 3D model was developed in order to model the vorticity on the quartz surface. The boundary conditions or the simulation are a turbulent inlet with atmospheric pressure and an outlet with a constant volume stream of 750 sccm.

**Figure 9.**
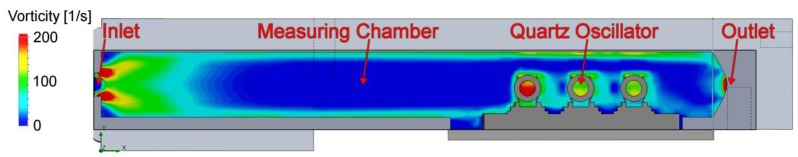
Vorticity of the air flow in the measuring tube with the quartz crystals. The inlet is defined with a constant atmospheric pressure of 1,024 mBar at 10% turbulences. The boundary condition of the outlet is a constant volume flow of 0.75 L/min.

Figure 9 displays the result of the numeric simulation. Impressively clear, the vorticity has its maxima at the inlet and at the location of the quartzes. The turbulences seem to build up a turbulent layer on the wall of the measurement chamber. This layer promotes vortices at the quartz surface. This will cause mechanical stress onto the quartz surface and most probably leads to noise in frequency.

#### 3.2.1. Laminar Flow Element

**Figure 10.**
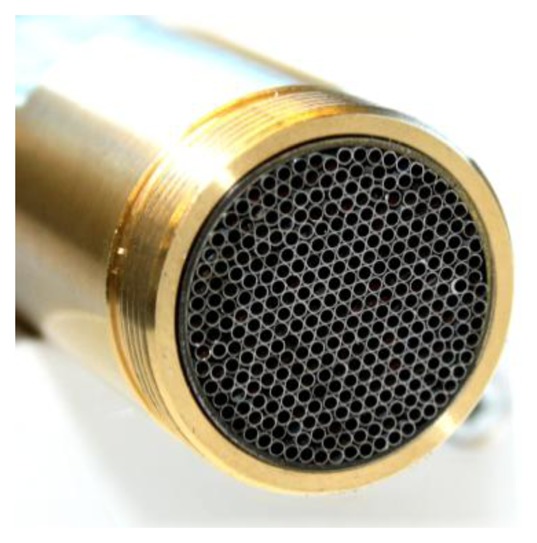
Laminar Flow Element with a set of 1 mm cannulas cut by wire EDM.

Reduction of the turbulent layer on the wall was tried by a laminar flow element (LFE) which removes turbulences at the inlet. The laminar flow element consists of a set of 1 mm cannulas cut by wire EDM (electrical discharge machining) as depicted in Figure 10.

**Figure 11.**
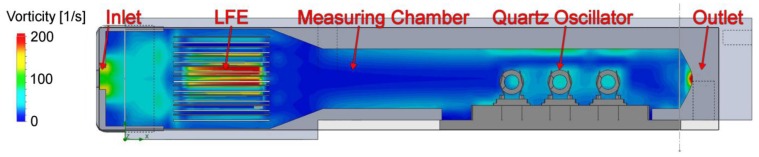
Vorticity of the air system in the measurement tube with laminar flow element. Inlet and outlet are defined with the same boundary conditions as in Figure 9.

**Figure 12.**
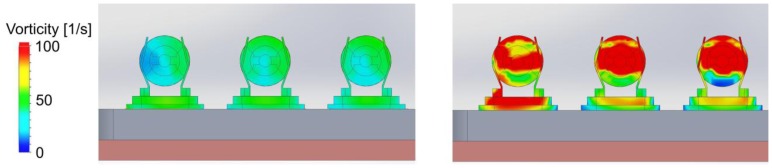
Detailed comparison of the vorticity at the quartz surface of Figures 6 and 7. The left simulation shows the vortex on the quartz surface of the measurement tube with LFE. On the right the simulation of the measurement system without LFE is displayed.

The simulation result of this set up is shown in Figure 11. The turbulences are located at the area between inlet and LFE but the air flow in the vicinity of the oscillators can be seen as laminar. A detailed view of the quartz loci in both simulations (Figure 12) illustrates that the vorticity at the quartz surface is tremendously reduced. For the evaluation of this concept, a removable LFE was designed probing the influence onto the measurement.

**Figure 13.**
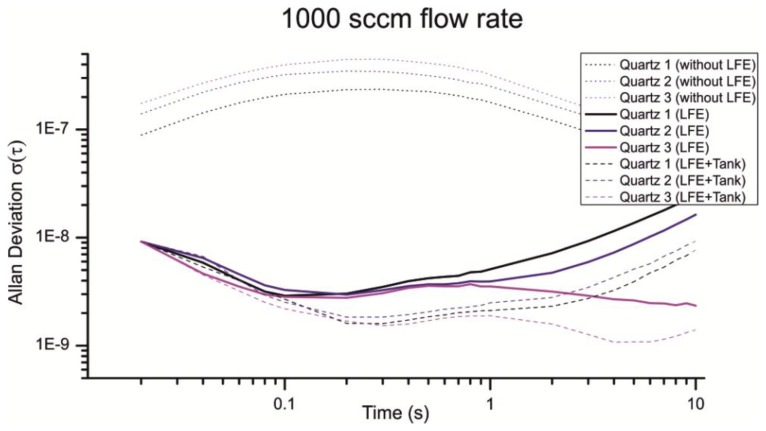
Comparison of different Allan deviations with a flow rate of 1,000 sccm. All measurement were taken at least 4 h after the device startup.

**Figure 14.**
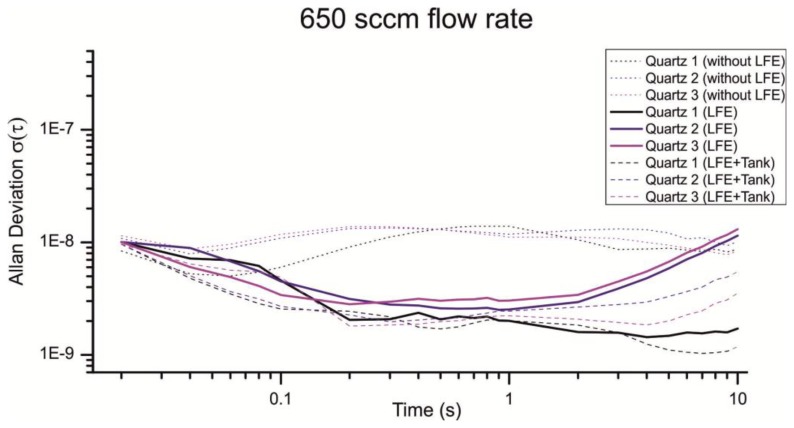
Comparison of different Allan deviations with a flow rate of 650 sccm. The Allan Devition was measured under the same conditions as Figure 13.

As depicted in Figure 13 the LFE reduces the noise introduced through turbulences considerably. From a flow rate of 1,000 sccm the Allan deviation is reduced from approximately 3 × 10^−7^ to 3 × 10^−9^ for a gate time of 200 ms. Interestingly the Allan deviation is just reduced for the half with a flow rate of 650 sccm, which is much fewer compared to a flow rate of 1,000 sccm (Figure 14).

Especially the Allan deviation of Quartz 1 with an air flow of 650 sccm (Figure 14, Quartz 1 LFE) indicates additional noise sources beside the 1/f noise, random walk and turbulences. For smaller flow rates the pump cycles are reduced, which lead to an irregular flow and a non-optimal load of the pump motor. An additional volume can be used to smooth the uneven airflow. Figures 13 and 14 show the resulting Allan deviation with an additional tank of ≈800 mL. The minimum Allan deviation is reduced to 2∼3 × 10^−9^ for both flow rates. Therefore the uneven air flow could be identified as another source in noise. However, the SNR can only be reduced a little which shows that a turbulent airflow is a major noise source. Compared to other existing QMB devices with an Allan deviation in the range of 10^−6^ to 10^−8^ we measured an excellent minimum Allan deviation τ(0.2s) ≈ 2∼3 × 10^−9^ with a constant air stream of 650–1,000 sccm.

### 3.3. Acceleration and Temperature Effects

The temperature behavior can be divided into static and dynamic temperature effects. The static temperature at the quartz crystal can dramatically affect the resonance frequency [22]. Nevertheless, this kind of temperature effect has just a small impact for QCM applications because no absolute frequency information is required.

#### 3.3.1. Static Temperature Effects

**Figure 15.**
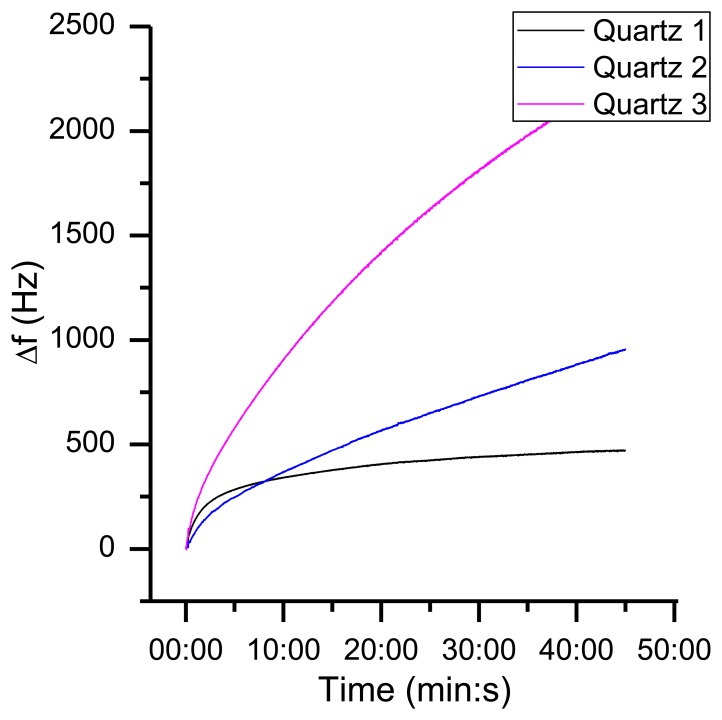
Frequency response upon warm-up with a constant air stream of 1,000 sccm.

When the resonator is powered, it takes some time before it reaches the thermal equilibrium (Figure 15). The length of this warm-up period depends on the electrical circuit, the input power and the thermal properties of the resonator. For example, a 195 MHz quartz crystal with a low Q-factor needs more power to oscillate compared to another 195 MHz quartz with a higher Q-factor. Therefore, the self-heating of the crystal will be larger. This is a reason why various quartz oscillators can have a different warm-up behavior. However, in the desired sensor application, the typical responses (*cf.* Figure 2) are on a much shorter time scale than this warming process and can be easily identified by mathematical filters.

**Figure 16.**
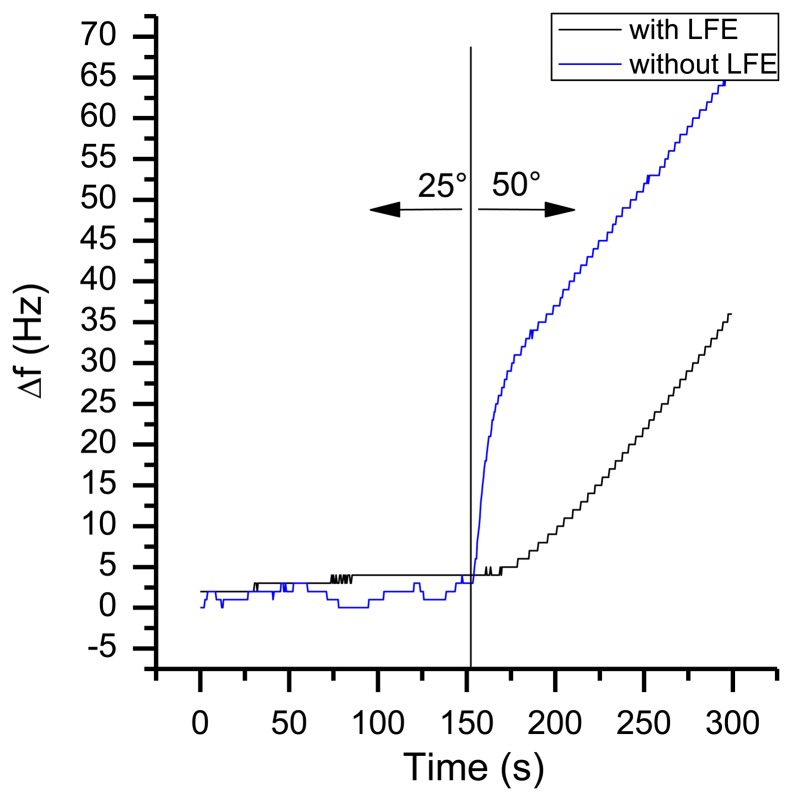
Frequency response to an environmental change of the temperature from 25 °C to 50 °C.

In a typical environment of a handheld sensor, temperature changes of more than 10 °C are possible. Figure 16 shows the frequency response of a changing environment. The sensor with an active pump was put from room temperature of 25 °C to an oven with 50 °C. As it can be seen, the sensor system with LFE shows just a small change in frequency with a maximum frequency slope of 1 Hz/s, whereas the system without LFE has a maximum slope of 3 Hz/s. Therefore the heat is equalized to the sensors temperature by passing the set of 1 mm cannulas. As a result, altering the absolute temperatur has just a small influence onto QCM measurements when using a heat compensation as the LFE. Like the warm-up behaviour, this kind of frequency shift can easily identified by mathematical filters.

#### 3.3.2. Dependency of Dynamic Temperature and Acceleration Effects

Aside of the described direct influences of temperature, the sensitivity towards acceleration effects seems to be strongly temperature dependent as well.

Acceleration effects on the frequency *f*(*a⃗*) are given by a function of direction and magnitude of the acceleration *a⃗⃗*. The dependency between acceleration and frequency is typically linear up to an acceleration of 50 g [22] and is given by [23,24]:

(4) $$f(\vec{a})=f_{0}(1+\vec{\Gamma }\cdot \vec{a})$$

The typical range of the sensitivity to acceleration | *Γ⃗* | (g sensitivity) can cover several orders of magnitude from 10^−7^ g for low cost crystals to 10^−10^ g for precision stress-compensated (SC) crystals [22]. It is caused by many factors as the quartz design, angle of cut, mounting structure, orientation, package type, and others [22–24]. All these effects cause mechanical stress, which will have an effect on the resonance frequency of the oscillator.

Physically, every expansion of particles will result in elongation *u⃗*, which will cause stress. For an infinitesimally small volume *δV* with a density of ρ the forces of a simplified resonator are given by Newton's Second Law of Motion *F_a_* and the mechanical stress per unit area *F_T_* [25]:

(5) $$F_{T}=\delta V\cdot \overset{3}{\underset{j=1}{\text{∑}}}\frac{\delta T_{ij}}{\delta x_{j}}$$

The local gradient of the mechanical stress *δT_ij_* is defined by the force in direction *i* and the surface with the normal *j*. Newton's Second Law of Motion can be rearranged to:

(6) $$F_{a}=m\cdot \vec{a}=\delta V\cdot \rho \cdot \vec{a}=\delta V\cdot \rho \frac{\delta \vec{u}}{\delta t^{2}}$$

Equalizing the formulas results in the following equation for the movement:

(7) $$\overset{3}{\underset{j=1}{\text{∑}}}\frac{\delta T_{ij}}{\delta x_{j}}=\rho \frac{\delta u_{i}}{\delta t^{2}}$$

Consequently, changing the mechanical stress tensor *δT_ij_* will also affect the resonant behavior of the quartz and therefore change its frequency. In a static environment, this stress will be constant for a specific quartz oscillator. As Kosinski explains the acceleration sensitivity causes a deformation of the crystal [26]. Zhou and Tiersten showed that by keeping the stress minimal and symmetric the sensitivity to acceleration can be reduced considerably [27].

**Figure 17.**
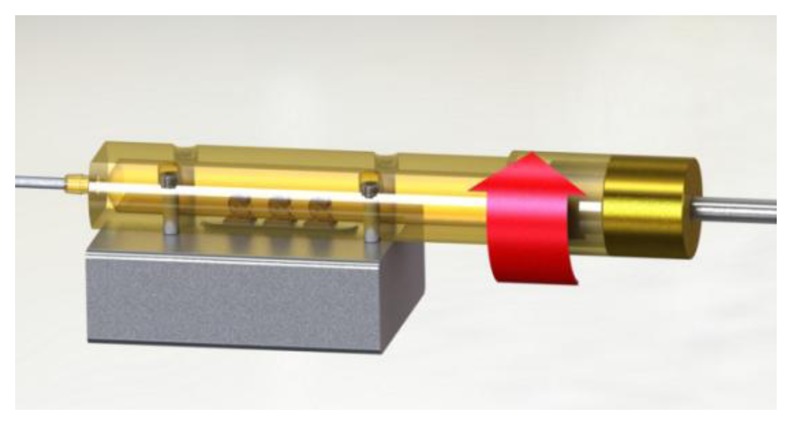
Clockwise rotation around the x axis of the sensor.

External forces, like gravity and a changing temperature gradient can change the mechanical stress which will result in a frequency shift. This is why a movement or rotation around the axis of the QCM sensor will cause an unwanted frequency shift. In standard quartz applications, the quartz oscillators are encapsulated and more robust, so the g sensitivity compared to the free-standing 195 MHz HFF-Quartzes must be lower. To test the g sensitivity, a special rotatable sensor module (without air flow inside) was rotated with a constant angular speed of 0.6 rpm around its x axis (Figure 17).

**Figure 18.**
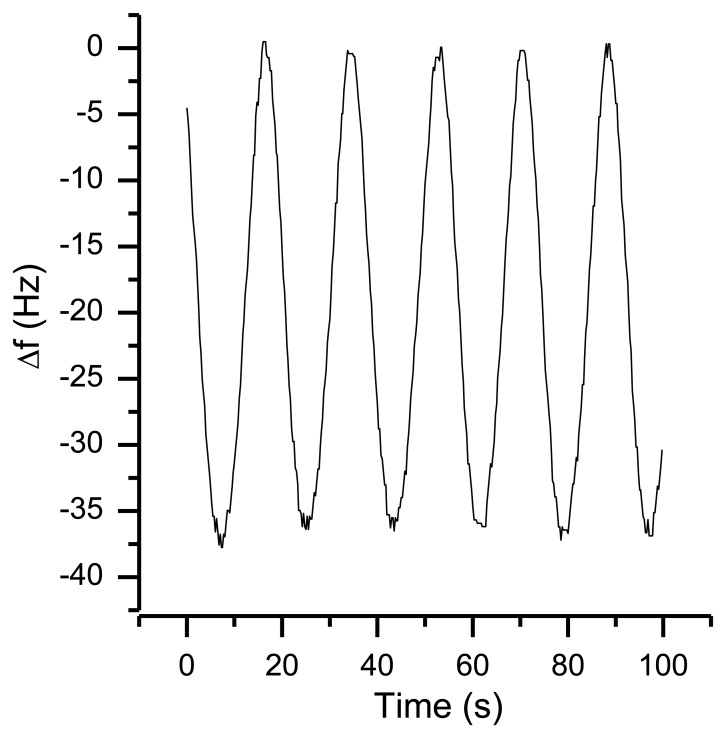
Frequency response of a rotation around the sensors x axis with a constant angular speed of 0.6 rpm.

Compared to gravity the centrifugal acceleration is negligibly small due to the small angular speed. As displayed in Figure 18, the rotation results in a frequency shift of more than 35 Hz. The same behavior was observed for rotations around the other axes.

**Figure 19.**
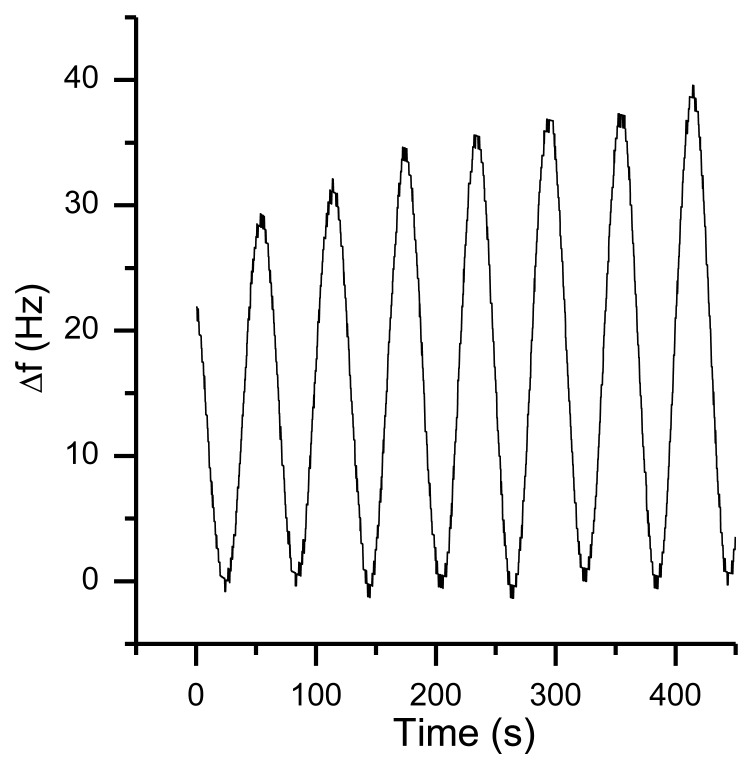
Frequency response of a rotation during the warm-up of the sensor.

Taking Equation (4) and a frequency shift of 35 Hz (as shown in Figure 18) into account, the value of the g sensitivity in direction of the x axis *Γ⃗_x_* is 9.2^−9^ g. For many applications, this is a reasonable result. However, in this setup, an orientation-dependent instability of 35 Hz reduces the resolution of the QCM dramatically. First indications that temperature effects might be involved in orientation-based signal shifts were observed when repeating the same experiment during the warm-up period of the oscillators (Figure 19). With a warming oscillator, the amplitude of the frequency changes increases as well.

**Figure 20.**
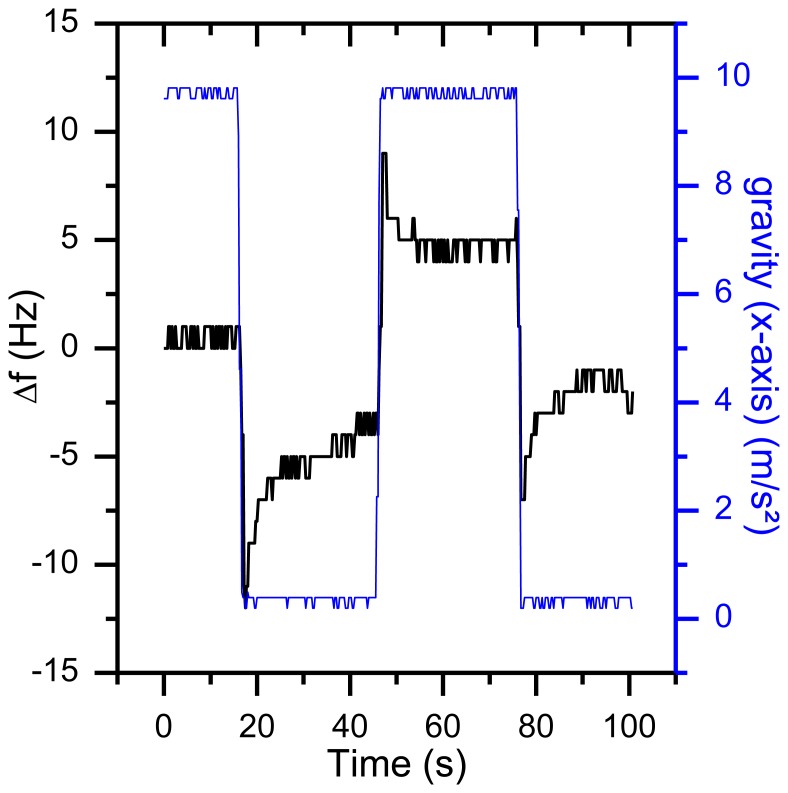
Frequency response of a 90° rotation around the sensors x axis.

**Figure 21.**
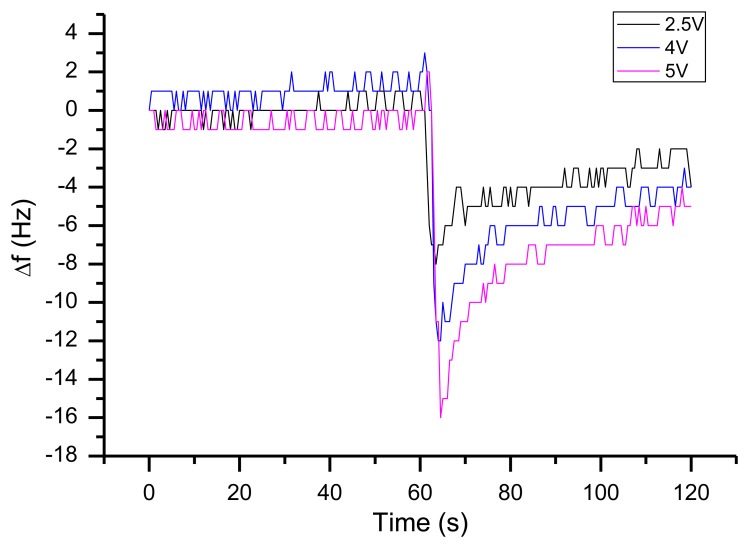
Frequency response of a 90° rotation with different supply voltages.

The result of tip-over experiment (a sudden 90° rotation around the x axis, no continuous rotation) can be seen in Figure 20. During the rotation the thermal gradient on the quartz surface seems to change, which results in an overshoot of the expected frequency shift. The most important heat source in this setup is the quartz oscillator itself. This was demonstrated by rising the supply voltage stepwise and repeating the tip-over experiment (Figure 21). With increasing voltage (and increasing temperature strain in the quartz) the g sensitivity increases as well.

**Figure 22.**
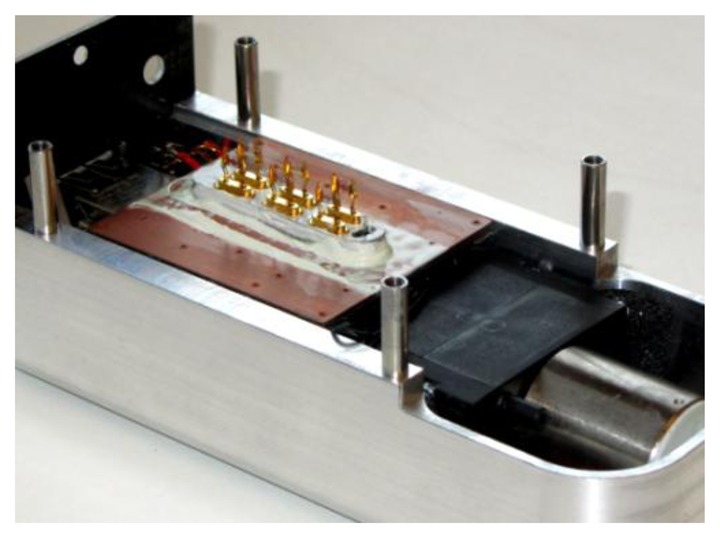
QCM sensor system without measuring chamber. A copper heat pipe and heat sink paste lead the heat from the oscillator off to the housing.

**Figure 23.**
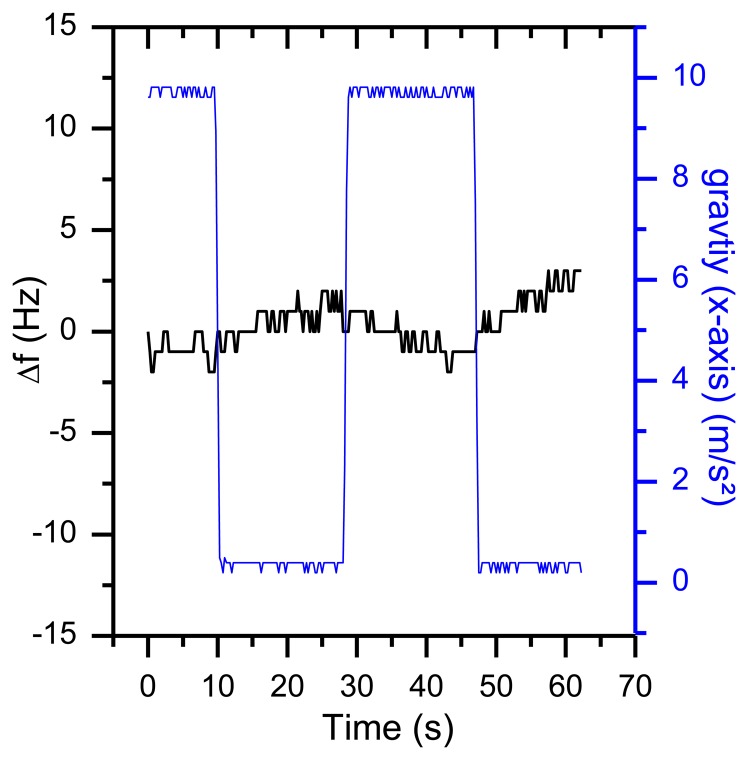
Frequency response of a 90° rotation with heat pipe at the oscillators.

All these results imply that temperature gradients inside the oscillator should be reduced in order to diminish mechanical sensitivity. Therefore, a heat pipe from the oscillators to the housing with heat sink paste was designed (Figure 22). Hence, the temperature around the quartz oscillators can be considered as constant. By doing this the unsymmetrical stress introduced by the temperature gradient of the crystal is reduced which leads to a reduction of the acceleration sensitivity (*cf.* [26–28]). With this knowledge, a QCM with a g sensitivity of less than 5–10 g could be achieved (Figure 23). Therefore, the sensitivity towards orientation changes is reduced by more than one decade simply by using a heat pipe from the oscillators and brings the system into close proximity to reference grade QCM with a acceleration sensitivity of 10^−10^ g.

## 4. Conclusions

Different design and layout optimizations for a continuously working QCM sensor in flowing air were described. Optimization of the electronic components yielded for a gate time in the range of 200 to 800 ms a standard deviation of 6 × 10^−10^ to 2 × 10^−9^. In order to compensate for “real-world” influences on the sensor stability, introduction of a laminar flow element and a heat pipe reduced the noise in frequency for a 195 MHz quartz to just 2∼3 × 10^−9^ in a mobile, handheld device. This will dramatically enhance the sensitivity for the detection of airborne analytes, e.g., traces of explosives. The application in the chemical sensing of volatile organic compounds will reported in due course.
